# Hemophagocytic Lymphohistiocytosis Associated With Parvovirus B19 in a Patient With Acquired Immunodeficiency Syndrome

**DOI:** 10.1177/2324709619883698

**Published:** 2019-10-21

**Authors:** Precious Macauley, Mohammad Abu-Hishmeh, Carissa Dumancas, Vijay Alexander-Rajan, Fernando Piedra-Chavez, Khaled Nada, Imnett Habtes, Andrea Popescu, Aleksandra Mamorska-Dyga

**Affiliations:** 1 Metropolitan Hospital Center, New York, NY, USA; 2 Lincoln Medical Center, New York, NY, USA

**Keywords:** hemophagocytic, lymphohistiocytosis, HIV, parvovirus

## Abstract

Hemophagocytic lymphohistiocytosis (HLH) is a rare and life-threatening condition characterized by widespread inflammation due to massive immune activation and cytokine release. It is of 2 types, primary or familial and secondary or acquired. Diagnosis is made by fulfilling 5 of 8 criteria as determined by the Histiocyte Society. Treatment includes etoposide, dexamethasone, with or without intrathecal methotrexate in the presence of neurologic involvement as well as treating the underlying cause in secondary HLH. We present a case of a 23-year-old female with congenital human immunodeficiency virus (HIV) infection who presents with nonspecific signs and symptoms of cough, fever, leukopenia, and anemia, and a high-serum parvovirus B19 DNA, later diagnosed with HLH and treated with etoposide and dexamethasone. She made clinical improvements and was successfully discharged to home after 26 days of admission.

## Introduction

Hemophagocytic lymphohistiocytosis (HLH) is a rare and life-threatening condition characterized by widespread inflammation due to massive immune activation and cytokine release.^1,2^ There are 2 types: primary and secondary. Primary HLH usually has a genetic predisposition and occurs in infancy, whereas the secondary or acquired form is usually triggered by infection, malignancy, or an autoimmune condition.^1-3^ The presentation is very nonspecific and includes fever, rash, cytopenias, lymphadenopathy, pulmonary dysfunction, hepatitis, meningismus, seizures, and septic shock.^1-3^

We report a case of parvovirus-associated HLH in a 23-year-old female with congenital human immunodeficiency virus (HIV) infection.

## Case Presentation

The patient is a 23-year-old African American female with congenital HIV infection and past infection with parvovirus B19 who presented with a 3-day history of high-grade fever and flu-like symptoms in the presence of neutropenia with white blood cell count 1.67 × 10^3^/L and anemia with hemoglobin 6.2 g/dL. She was known to be intermittently compliant with antiretroviral therapy. Management of febrile neutropenia was commenced with broad-spectrum antibiotics. However, the following day, she developed septic shock despite adequate fluid resuscitation and was transferred to the intensive care unit. Treatment continued with broad-spectrum antibiotics, antifungals, and 2 vasopressors for presumed septic shock. The patient developed profuse watery diarrhea for which infection with *Clostridium difficile* was suspected but later ruled out by a negative stool analysis by stool polymerase chain reaction.

An extensive microbiologic workup was undertaken including bacterial, viral, and fungal cultures and serology. An autoimmune etiology was ruled out by negative antinuclear and anti-mitochondrial antibody, low C3 level, and normal C4 level. Her CD4 count was found to be 82 cells/µL. Parvovirus B19 DNA was markedly elevated (see Table 1), as well as a positive immunoglobulin M (IgM) and negative IgG for parvovirus B19. She was started on dexamethasone and intravenous immunoglobulin (IVIG) for the management of aplastic anemia secondary to parvovirus B19 infection.

**Table 1.** Laboratory Tests.

Laboratory Values (Unit)	Normal Range	Day 1	Day 7^a^	Day 14	Day 60
WBC (/nL)	4.3-11	1.67	0.48	0.09	8.23
Hb (g/dL)	12-16	6.2	5.9	8.5	11.3
Platelets (/nL)	238	448	39	13	238
Creatinine (mg/dL)	0.7-1.2	0.6	3.2	4.5	1.3
Urea (mg/dL)	6-20	10	50	133	31
Haptoglobin (mg/dL)	34-200	172	NA	NA	NA
INR	0.8-1.14	1.03	1.01	1.03	0.89
PTT (seconds)	28-37	21	104	24	22.6
Ferritin (ng/mL)	15-150	NA	82 537	2201	860
LDH (Units/L)	100-190	155	1319	861	240
Triglycerides (mg/dL)	10-149	NA	397	171	113
Fibrinogen (mg/dL)	200-400	276	260	295.8	519
CD25 (pg/mL)	≤1033	NA	12 080	NA	NA
Parvovirus PCR (IU/mL)	Negative	9.90E + 09	NA	33 100	4400
ESR (mg/dL)	0-20		>100		
CRP (mm/h)	0-0.4		10.92		

Abbreviations: WBC, white blood cell count; Hb, hemoglobin; NA, not available; INR, international normalized ratio; PTT, partial thromboplastin time; LDH, lactate dehydrogenase; PCR, polymerase chain reaction; ESR, erythrocyte sedimentation rate; CRP, C-reactive protein.

a Day 7 is the day hemophagocytic lymphohistiocytosis criteria was fulfilled and therapy started.

Concurrently, the patient was continued on broad-spectrum antibiotics, which included antibacterial, antifungal, and antiretroviral agents. However, the patient’s medical condition continued to deteriorate developing acute tubular necrosis, liver failure, and rhabdomyolysis. Her mental status worsened requiring intubation and mechanical ventilation on the fifth hospital day, and meningitis was eventually ruled out by cerebrospinal fluid analysis.

**Figure 1.**
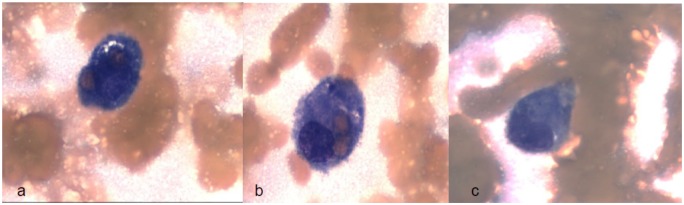
Histology of bone marrow biopsy.

Further investigations recommended by the hematology team revealed an elevated triglyceride and ferritin level (Table 1). The constellation of findings that included fever, pancytopenia, hypertriglyceridemia, and hyperferritinemia increased the suspicion for HLH. The diagnosis was confirmed on the seventh hospital day by bone marrow biopsy and aspirate that showed evidence of hemophagocytosis (Figure 1). A cytokine panel that included soluble CD25 receptor (sCD25r) and interleukin-2 levels were also sent but and later showed a high sCD25r of 12 080 pg/mL (normal <1033 pg/mL), further supporting the diagnosis of HLH.

Etoposide (150 mg/IV) with high-dose dexamethasone (20 mg/IV) infusion was immediately started after confirming the HLH diagnosis by bone marrow biopsy and aspirate. She also received 2 more doses of IVIG after the diagnosis of HLH. Her condition steadily improved after 48 hours of starting the etoposide, marked by defervescence, improvement of mental status, liver function panel, rhabdomyolysis, and subsequent extubation on the 11th hospital day. The second dose of the etoposide was delayed 2 weeks from the first one due to the pancytopenia and renal function. She received IVIG for a total of 5 days (2 doses prior to and 3 doses after the diagnosis of HLH), and dexamethasone was tapered over a 2-week period. The patient refused to be transferred to the inpatient rehabilitation unit for management of critical illness myopathy. However, she continued to follow-up at the hematology and HIV clinic, and her clinical status remained stable hence did not require additional doses of etoposide.

## Discussion

Hemophagocytic lymphohistiocytosis is a very serious life-threatening condition with a very high mortality rate if not treated promptly.^1,2^ As a consequence of the intense inflammatory state brought about by cytokine release and the nonspecificity of symptoms, most patients are treated with broad-spectrum antibiotics for presumed sepsis before finally arriving at the diagnosis.^3^ The secondary or acquired form is more common in adults who have an acquired defect in lymphocyte and natural killer cell cytotoxic function as in our patient who has acquired immune deficiency syndrome (AIDS).^1-3^

The triggers in these patients could be infectious, malignant, or autoimmune.^1,3^ Natural killer cells and T-cells cause apoptosis of antigen-presenting cells and infected cells by perforin- and granzyme-dependent pathways.^1-3^ These aforementioned cells also contain and downregulate the immune response generated by the perforin- and granzyme-mediated killing; mutations in these pathways lead to both defective apoptosis and hyperactivation, resulting in intense cytokine release by these cells and is the proposed mechanism in primary HLH.^2,3^ In secondary HLH, the exact mechanism is not known; however, regardless of the etiology, the final effect is massive release of cytokines resulting in the clinical and laboratory findings.^1,3^ Cytokine release drives proliferation of macrophages leading to phagocytosis of leukocytes, platelets, erythrocytes, and their precursors in the reticuloendothelial system.^2,3^ Fever occurs as a result of release of tumor necrosis factor-α (TNF-α) and interleukins. Hypertriglyceridemia becomes evident due to the cytokine-induced inhibition of lipoprotein lipase. Macrophages release ferritin causing activation of plasminogen, which causes enhanced lysis of fibrinogen manifesting in hypofibrinogenemia.^1-3^

A host of triggers have been reported in HIV-positive individuals including viral, protozoan, fungal, and bacterial infections including acute HIV seroconversion itself as well as T- and B-cell lymphomas, drugs, and autoimmune disorders.^1-5^ Epstein-Barr virus has been the most commonly reported cause of secondary HLH both in HIV and non-HIV individuals.^2-4^ In our patient, we believe that reactivation of latent parvovirus B19 infection was the causative factor. This virus is one capable of switching between a lytic phase (active viral replication) to a latent (dormant) phase. The switch from a dormant to a lytic state is known as reactivation, a process that can be triggered by other viral infections, physiologic stressors, and immunosuppression, though sometimes a trigger is not apparent.^5^ As our patient was intermittently compliant with antiretroviral therapy, worsening immunosuppression is likely the trigger for reactivation of parvovirus as her CD4 count was 82 cells/µL. Diagnosis of parvovirus B19 infection can be challenging in patients with severe immunocompromise as they are usually unable to mount neutralizing antibodies to clear the virus, and serology for anti-parvovirus B19 is usually negative or show weak titers of IgM alone.^6^ This was the case of our patient; despite her prior infection, her serology was only positive for anti-parvovirus IgM and negative for anti-parvovirus IgG. Of note, the patient’s CD4 count was 6 during the previous admission, the time at which she was first diagnosed with parvovirus B19 infection.

Although parvovirus B19 is not one of the more commonly reported viral associations with secondary HLH, cases do exist in both HIV and non-HIV seropositive individuals.^3,7,8^ One report suggests that parvovirus-triggered HLH may have more favorable outcome than other infectious triggers.^7^

As mentioned in Table 2, the diagnosis of HLH is made when 5 out of the 8 criteria are met; however, in HIV-infected individuals, hemophagocytosis may not be readily evident and repeat testing may be necessary.^3,8^ Treatment should be started promptly considering that HIV-associated HLH can be more fulminant and aggressive with higher mortality rates in those with CD4 counts less than 200 cells/µL.^3,4^

**Table 2.** Hemophagocytic Lymphohistiocytosis Diagnostic Criteria Adapted From Henter et al.^9^

Criteria for Hemophagocytic Lymphohistiocytosis Diagnosis^a^	Criteria Met by Our Patient
Fever (>38.5°C)	Yes
Splenomegaly	No
Pancytopenia (at least 2 cell lineages must be affected); hemoglobin <9 g/mL, platelets<100 000/mm^3^, neutrophils <1 × 10^9^ L/min	Yes
Ferritin 500 ng/mL or more	Yes
Hemophagocytosis in the bone marrow, spleen, or lymph nodes	Yes
Low or absent natural killer cell activity	No
Hypertriglyceridemia (265 mg/100 mL or more) and/or hypofibrinogenemia (150 mg/100 mL or less)	Yes
Elevated soluble IL-2 receptor (>2400 U/mL) or >2 standard deviations above the age-adjusted mean	Yes

a Five of 8 clinical criteria must be present for diagnosis.

There are 2 major protocols investigating therapy for HLH, one performed in 1994 and the second in 2004 by the Histiocyte Society. The HLH-94 protocol included etoposide and dexamethasone for 8 weeks followed by a continuation phase that added daily oral cyclosporine A. Dexamethasone is the corticosteroid of choice due to its increased ability to cross the blood-brain barrier.^9-11^ Intrathecal methotrexate is indicated in patients with progressive neurological decline.^9-11^ The HLH-2004 protocol differed from the HLH-94 protocol by the addition of cyclosporine to the initial therapy with the aim of decreasing pre-hematopoietic stem cell mortality.^11^ This latter approach failed to show improvement in HLH outcome; however, this study did confirm the efficacy of etoposide and cyclosporine.^9,11^ Hematopoietic stem cell transplantation is indicated in patients with familial, recurrent, or relapsing HLH.^8,11^ These agents work by inhibiting macrophage activity and thereby decreasing the production of cytokines.^8-11^ It should be noted that these studies included individuals younger than 18 years of age, which included both confirmed familial or primary HLH and those without a family history. Presently, it is rare to include cyclosporine A in the treatment of secondary HLH, although there is some evidence of its efficacy in young adults with Epstein-Barr virus–associated HLH.^10^ Other agents have been added to the regimen depending on the underlying associated condition; for example, rituximab has been used in diffuse large B-cell lymphoma–associated HLH.^10^ In our patient, a subsequent course of IVIG was added to the regimen after the diagnosis of HLH. While IVIG has been used successfully in the treatment of parvovirus-associated HLH, there are some cases that have been treated with high-dose corticosteroids alone.^8,12^ We believe that in our patient, IVIG alone was insufficient in treating HLH, and etoposide and dexamethasone were required as the patient only began showing clinical improvement with the initiation for the latter 2 agents. Regardless, the underlying cause must be sought after and treated if possible, similar to our patient’s worsening immune suppression, which was corrected with initiation of antiretroviral agents.^3,4,8,9^

## Conclusion

Hemophagocytic lymphohistiocytosis has a very high mortality rate invariably culminating in fatality without medical intervention. For this reason, a high index of suspicion should be maintained in individuals who present in as unexplained hyperinflammatory state.
